# How to Recognize and Treat Small Intestinal Bacterial Overgrowth?

**DOI:** 10.3390/jcm11206017

**Published:** 2022-10-12

**Authors:** Barbara Skrzydło-Radomańska, Bożena Cukrowska

**Affiliations:** 1 Department of Gastroenterology, Medical University of Lublin, Jaczewskiego 8, 20-950 Lublin, Poland; 2 Department of Pathomorphology, The Children Memorial Health Institute, Aleja Dzieci Polskich 20, 04-730 Warsaw, Poland

**Keywords:** SIBO, microbiota, dysbiosis, rifaximin, probiotics

## Abstract

Small Intestinal Bacterial Overgrowth (SIBO) is a form of dysbiosis that involves increased bacterial colonization of the small intestine with some of the bacteria more characteristic of the colon microbiota. The prevalence of SIBO over recent decades has been estimated to range from 2.5 to 22% (depending on the source) and to increase with age and among individuals with comorbidities. Recently, an increase in the number of diagnosed SIBO cases has been observed, which is primarily due to the availability of noninvasive breath tests that facilitate the diagnostic process. However, SIBO is still both a diagnostic and a therapeutic problem. This review presents the pathophysiology, manifestations, diagnostics, and recommended management of SIBO.

## 1. Introduction

The medical phenomenon currently known as Small Intestinal Bacterial Overgrowth (SIBO) was identified several decades ago by Faber, who described it in 1897 in the form of a case report of ‘blind-loop syndrome’, with that term also used in some subsequent papers on this topic [1,2]. Currently, SIBO is defined as a form of dysbiosis characterized by increased numbers of bacteria colonizing the small intestine, possibly with some characteristics of the colon microbiota [3,4,5]. The hallmark of SIBO is this excessive number of bacteria, both aerobic and anaerobic, within the small intestine, which is usually only sparsely colonized. The number of bacteria can be accurately determined via cultures of the fluid endoscopically aspirated from the small intestinal lumen. This diagnostic technique helped establish a quantitative definition of SIBO, which—initially—was defined as the number of bacteria above 10^5^ colony forming units (CFU) per milliliter of the aspirate collected from the third part of the duodenum on upper endoscopy [3]. Currently, the cut-off for SIBO diagnosis is above 10^3^ CFU/mL of aspirate [5,6,7].

It is worth noting that the forms of SIBO differ depending on the predominant species of bacteria colonizing the small intestine, and these depend on their origin. There are two well-established types: upper aerodigestive tract (UAT) SIBO and coliform SIBO [8]. UAT SIBO is caused predominantly by oral cavity bacteria, including *Prevotella* sp. and *Streptococcus viridans,* whereas coliform SIBO is characterized predominantly by bacteria from the distal segments of the gastrointestinal tract, such as *Escherichia coli*, *Klebsiella pneumoniae*, *Proteus mirabilis*, *Enterococcus* sp., or *Clostridioides* sp. [8,9]. Despite the distinction between the two types, their importance in clinical practice is limited due to the similar symptoms and treatment.

The problem of SIBO has been steadily gaining attention since the first half of the 2000s. However, controversies remain as to its diagnostics and management. This review presents the most recent data on epidemiology, the pathological mechanism, clinical presentation, and recommended diagnostic and therapeutic management of SIBO.

## 2. The Prevalence, Pathophysiology, and Risk Factors of SIBO

The exact prevalence of SIBO in the general population is unknown. Most authors report rates between 2.5 and 22% and emphasize that the prevalence increases with age and in populations with comorbidities [3,6,10]. The discrepancies in the reported prevalence rates result from the fact that SIBO syndrome may be asymptomatic or manifest with symptoms (such as abdominal bloating, pain, or altered bowel habits) that may be caused by other factors. Moreover, the clinical presentation of SIBO is a common epiphenomenon accompanying a number of systemic diseases (such as diabetes) or diseases of other organ systems (such as connective tissue disorders) and is not immediately considered in the differential diagnosis.

There are many protective mechanisms to ensure small intestinal microbiota homeostasis and prevent excessive bacterial colonization. When these mechanisms are overwhelmed, insufficient, or absent, this may lead to SIBO. There are three types of protective mechanisms against SIBO [3,4,6]:

1. **Gastrointestinal antimicrobial defense mechanisms** connected with the production of hydrochloric acid (which may be deficient due to atrophic gastritis, long-term proton pump inhibitor use, or gastric resection), pancreatic enzyme secretion (impaired in chronic pancreatitis or cystic fibrosis), bile, and mucosal immunity (compromised in AIDS or insufficient immunoglobulin A secretion);
2. **Gastrointestinal motility.** Motility disorders impair small intestinal clearance due to the absence of phase III of the migrating motor complex and retrograde peristalsis. Such disorders may be due to primary visceral neuropathy or myopathy (intestinal pseudo-obstruction) or, much more commonly, secondary neuropathy (e.g., in diabetes; amyloidosis; scleroderma; Parkinson’s disease; iatrogenic effects of drugs, such as opioids or anticholinergic and antidiarrheal agents);
3. **Gastrointestinal tract anatomy.** Anatomical anomalies include small intestinal diverticuli, strictures, adhesions, and interloop fistulae; secondary structural anomalies, which are often a result of Crohn’s disease, radiotherapy, or surgical interventions (gastrojejunostomy, colectomy, or ileocecal valve resection).

Other risk factors for SIBO are age (the risk increases with age), female sex, and concomitant diseases of other organs and systems; apart from the comorbidities listed above, these include irritable bowel syndrome (IBS), celiac disease, and liver diseases [4,6,11,12]. Of note is the frequent coexistence of SIBO and IBS. SIBO rates in patients with IBS are estimated to be up to seven times higher (ranging from 51.7 to 78%) than in individuals without IBS [13,14]. 

## 3. Clinical Presentation of SIBO

**Figure 1:**
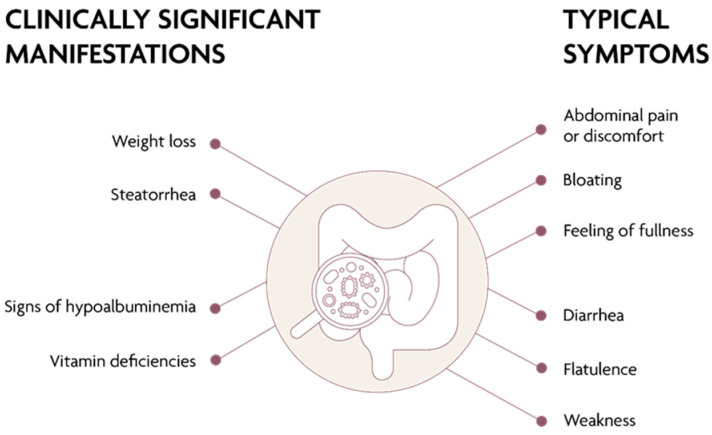
Clinical presentation of SIBO.

SIBO may cause various clinical manifestations as well as have an asymptomatic course. The most common symptoms, reported by two-thirds of patients with SIBO, are abdominal distension, excessive gas accumulation and flatulence, the feeling of abdominal fullness, diffuse abdominal cramps, and altered bowel habits (predominantly diarrhea or alternating bowel habit, sometimes constipation) (Figure 1). The patients may also complain of chronic fatigue and impaired concentration [4,10,11]. Constipation accompanies SIBO when bacterial overgrowth is primarily due to methane-producing (methanogenic) microorganisms, such as *Methanobrevibacter smithii* [15]. Notably, methane is produced mainly by *Archaea*—the domain of microorganisms, distinct from bacteria. Consequently, the word “bacterial” in the original name of this condition (“methane SIBO”) posed a terminological conflict, which has been resolved by the introduction of the new term intestinal methanogen overgrowth (IMO) to express the diagnosis in patients with a positive methane breath test [11,16]. 

In more severe cases of SIBO, the diarrhea may be fatty (steatorrhea), which leads to weight loss and undernutrition. Malabsorption may include fat-soluble vitamins, such as vitamin A, D, and E, but also vitamin B12 and iron—with the resulting micro- or macrocytic anemia, polyneuropathy, and bone metabolism disorders. Most patients have no deficiencies of folic acid or vitamin K, since these are products of bacterial metabolism [3,10,11,17]. 

Due to the lack of specific symptoms, the diagnosis of SIBO requires comprehensive diagnostic assessments. The fact that SIBO commonly co-occurs with other pre-existing conditions may pose a further hurdle to its effective treatment or even exacerbate the symptoms [14,18,19]. Conversely, SIBO eradication may lead to therapeutic success for comorbidities. It is particularly important to consider the co-existence of SIBO in patients with chronic pancreatitis, cystic fibrosis, celiac disease seemingly resistant to treatment with a gluten-free diet, inflammatory bowel disease flare-ups (particularly in patients with Crohn’s disease), exacerbated symptoms following ileocecal valve resection, symptoms of decompensated liver disease with ascites at risk of spontaneous bacterial peritonitis, or IBS. Bacterial overgrowth eradication in these situations may constitute a major therapeutic goal. In fact, the Polish Gastroenterology Association guidelines recommend targeted diagnostics for, and eradication of, SIBO in managing patients with diarrhea-predominant and mixed IBS [20]. One undoubtedly important and recommended step in SIBO management is to determine the underlying cause since causative treatment is likely to reduce the risk of SIBO recurrence.

## 4. SIBO Diagnostics

The presence of clinical manifestations indicative of SIBO is not sufficient grounds for introducing eradication treatment with systemic antibiotic therapy. Any such treatment must always be preceded by diagnostic assessments [11,16]. Physical examination in patients with SIBO typically reveals no marked abnormalities. Some patients have considerable abdominal distension. Palpation may reveal segmentally constricted or distended intestinal loops. Laboratory test abnormalities may be found in more severe forms of SIBO and typically include megaloblastic anemia, iron-deficiency anemia, fat-soluble vitamin (A, D, and E) deficiencies, vitamin B12 deficiency, and hypoalbuminemia [2,3,6,10,11]. Endoscopy of the upper and lower gastrointestinal tract and histology of small intestinal biopsy samples show no pathological changes or non-specific increase in the number of intraepithelial lymphocytes, especially gamma/delta positive T cells [21,22]. This unique T-cell population might have a key role in the defense against intestinal bacterial pathogens [22]. Since intraepithelial lymphocytosis with the correct architecture of intestinal villi can also occur in celiac disease (so-called potential celiac disease), serological testing of specific celiac antibodies should always be performed in this case [21].

Cultures of endoscopically obtained small intestinal (or—most commonly—distal duodenal) aspirates are considered to be the ‘gold standard’ in SIBO diagnostics [11,23]. The most important limitations of this method are the lack of standardized techniques of aseptic sample collection to eliminate the risk of sample contamination and the time-consuming nature, invasiveness, and cost of this technique. Alternatively, to traditional culture-based methods, microbial identification can be achieved via genetic 16S ribosomal RNA PCR-based analysis [24]. Nonetheless, these methods are not used for diagnosing SIBO in everyday practice; instead, they are used for research purposes and for complex differential diagnostics. 

Currently, breath testing as a safe and non-invasive technique is used in SIBO diagnosis [11,15,25]. Breath tests measure the concentration of such gases as hydrogen, methane, or possibly hydrogen sulfide (although validation is still needed in the case of this last gas) in the exhaled air following oral administration of lactulose or glucose. Breath testing is based on the fact that human body cells do not produce the gases that are being measured, and increased levels of those gases in the exhaled air indicate increased metabolism of undigested carbohydrates that undergo fermentation by the microorganisms colonizing the small intestine. Breath testing used in diagnosing SIBO is noninvasive, accessible, and cost-effective, though its sensitivity and specificity are limited. A recent Italian meta-analysis of 14 clinical studies showed that depending on the substrate used—either lactulose at 10 g or glucose at 75 g—the sensitivity was 42% and 54.5%, and specificity was 70.6% and 83.2%, respectively [25]. The diagnostic concordance between small intestinal aspirate culture and routinely used breath testing is approximately 65% [23]. Considering the results described the usefulness of the breath tests at present seems limited.

It is crucial to follow the guidelines for preparing a patient for a breath test (Table 1) [26,27]. In the case of patients on antibiotics or probiotics, the test can be performed only after at least a 4-week washout period. In cases of infectious diarrhea or long-term probiotic use, the test may be performed after at least two weeks have passed; proton pump inhibitors must be categorically discontinued a week before the breath test. Any drugs that affect intestinal motility (e.g., loperamide, metoclopramide), even short-acting ones, should be discontinued two days before the test. During the last 24 h before the test, the patient should not consume any alcohol or dietary fiber. Breath testing should be performed after a 12-h fast, and the patient should avoid exertion and refrain from smoking on the day of the test.

According to American guidelines, the result required for an SIBO diagnosis in a hydrogen breath test is an increase in exhaled hydrogen concentration by at least 20 parts per million (ppm) from baseline within 90 min of the initial measurement after oral administration of the substrate [11,16]. Any increase in hydrogen concentration after 90 min reflects colonic fermentation of the administered substrate. In methane breath testing, a cut-off value of 10 ppm at any time point during the test indicates IMO, since methanogen overgrowth may involve both the small and large intestine [6,11,16]. In patients with IMO, the sensitivity of a hydrogen breath test may be diminished, irrespective of whether lactulose or glucose is used as the substrate, since the hydrogen produced by bacterial fermentation may be used for the production of methane and hydrogen sulfide. Therefore, in order to increase the accuracy of the test, both hydrogen and methane concentrations should be measured in the exhaled air [16]. It is important to bear in mind that the sensitivity and specificity of these breath tests are limited not only for the reasons mentioned above. Breath tests with lactulose yield up to one-third of false positive results since the osmotically active lactulose may increase intestinal motility and cause the measurement at the pre-specified 90 min time point to reflect not the fermentation due to the microorganisms colonizing the small intestine but that caused by the colonic microbiota [27]. When glucose is the substrate, it may be absorbed in the proximal jejunum and never reach its distal segment (which is colonized as a result of SIBO), consequently failing to produce the expected peak in the measured exhaled hydrogen levels. Additionally, glucose must not be used as a substrate for breath testing in diabetic patients.

## 5. SIBO Treatment

The primary goal of SIBO treatment is to eradicate microorganisms from the small intestine in order to reduce symptoms. Further treatment goals are to maintain remission, prevent possible recurrences, and correct any nutritional and vitamin deficits. Apart from improving the effects of treating the underlying condition (diabetes, cystic fibrosis, and pancreatic insufficiency) and possibly correcting the unwanted effects of prior surgical treatment, SIBO management may involve diet therapy. 

### 5.1. Diet 

A diet low in fermentable oligosaccharides, disaccharides, monosaccharides, and polyols, the so-called low FODMAP diet—which is also used in IBS—deprives bacteria of their source of energy necessary for proliferation and reduces bacterial fermentation, as evidenced by low levels of hydrogen in breath tests [28,29]. It is worth emphasizing that the period of complete elimination of FODMAP from the diet of SIBO patients should not exceed six weeks; and, if ineffective, this diet should not be used again in the future [11,30]. There is no evidence supporting the use of a gluten-free diet in the treatment of either SIBO or IBS [28]. The use of elemental diets, which contain pre-digested nutrients, is not recommended in SIBO despite some promising study reports [6,31].

### 5.2. Antibiotic Therapy

According to the North American Consensus from 2020, oral antibiotics play a key role in SIBO eradication [11]. The purpose of antibiotic therapy is not to eradicate completely (which is not always possible) the bacteria colonizing the small intestine but to modulate the small intestinal microbiota in a manner that leads to improvement of SIBO symptoms. Most studies on SIBO treatment have evaluated the efficacy of amoxicillin with clavulanic acid, ciprofloxacin, doxycycline, metronidazole, neomycin, norfloxacin, tetracycline, co-trimoxazole, or rifaximin [32]. In light of the lack of large randomized clinical trials evaluating the effects of antibiotics in the treatment of SIBO, antibiotic therapy is usually empirical. In addition, there are limited data comparing the efficacy of different antibiotics. Another problem with antibiotic therapy is the need to repeat antibiotic treatment due to a recurrence of SIBO. Lauritano et al. showed SIBO symptom recurrence in 12.6%, 27.5%, and 43.7% of patients three, six, and nine months after successful treatment, respectively [33]. It should be emphasized that antibiotic re-treatment may be associated with an increased risk of antibiotic resistance, diarrhea, including *Clostridioides* infection, intolerance, and gut microbiota dysbiosis [32]. 

Recently, rifaximin has been increasingly used in the treatment of SIBO [34,35]. Rifaximin has a good safety profile, is not absorbed from the gastrointestinal tract, dissolves well in bile, has broad-spectrum antibacterial effects against Gram-positive and Gram-negative aerobic and anaerobic bacteria, and its side effects are comparable with those of a placebo. What is particularly noteworthy is the fact that, according to studies, rifaximin acts like a eubiotic in the gastrointestinal lumen, which means that it protects the intestinal microbiota and increases the number of beneficial bacterial strains of the genera *Lactobacillus* and *Bifidobacterium,* reduces inflammation, augments intestinal barrier function, and limits bacterial translocation [36]. Another valuable characteristic of rifaximin is the fact that it does not produce bacterial resistance, and therefore, can be used again (provided the necessary interval of at least four weeks is maintained between consecutive 14-day courses of rifaximin treatment) [37]. Two recent meta-analyses assessing the safety and efficacy of rifaximin in the treatment of SIBO demonstrated high rates of successful eradication (71% and approximately 60% in the meta-analyses from 2017 and from 2021, respectively) [34,35]. 

### 5.3. Probiotics

Probiotics are live microorganisms that, when administered at appropriate doses, exert beneficial effects on the host’s health [38]. In the case of SIBO, the importance of probiotics is mostly due to their ability to modulate the composition of intestinal microbiota and protect the gut against colonization by pathogens. Probiotics are a source of a number of substances that limit the growth and metabolism of microorganisms, including commensal bacteria. They produce antimicrobial substances, such as anti-microbial peptides, bacteriocins, reuterin, and numerous metabolites (e.g., lactic acid), all of which negatively affect the proliferation of gut-colonizing microorganisms [39,40]. Moreover, probiotics compete with other bacteria for nutrients and adhere to the gut epithelium [41]. Because of these properties of probiotics, there have been attempts to augment SIBO treatment with probiotic strains exhibiting clinically proven effects [41,42,43,44,45]. Although most studies so far were conducted in small groups of patients and evaluated different endpoints, a 2017 meta-analysis of probiotic therapy in SIBO showed that the use of probiotics reduces the hydrogen produced in breath testing (odd ration (OR) = 1.61, 95% of the confidence interval (Cl) = 1.19–2.17) [46]. 

It is important to remember that, like in the case of other indications, the effects of probiotic therapy in SIBO are strongly strain-dependent, and not all probiotics are equally effective [38]. A study from 2018 showed that probiotic administration in SIBO patients resulted in exacerbated bloating, flatulence, metabolic lactic acidosis, and “brain fog,” with these symptoms subsiding after the probiotic preparation was discontinued and antibiotic therapy was initiated [47]. Therefore, probiotic therapy should be carefully considered in each individual case, as it may either bring the expected improvement or exacerbate the existing condition. Moreover, data from clinical studies indicates that the use of probiotics for one month may predispose to IMO [6]. 

There is still no clear consensus on the issue of probiotic therapy in SIBO, which means that further well-designed clinical studies are needed to establish the role of probiotics in the therapeutic management of patients with SIBO. This conclusion can be drawn from the American guidelines on SIBO treatment [11]. Therefore, our interdisciplinary research team attempted to assess the effects of single-strain probiotic preparations containing a *Bifidobacterium lactis* strain (NORDBIOTIC™ BI040) or a *Bacillus coagulans* strain (NORDBIOTIC™ BC300) on clinical symptoms and the concentration of hydrogen in a breath test in patients with IBS and SIBO. This randomized, placebo-controlled trial was registered with the number NCT05064930 at ClinicalTrials.gov. The trial is scheduled to be completed in December 2022. 

## 6. Conclusions

SIBO is characterized by increased numbers of bacteria in the small intestine associated with a set of symptoms, including bloating, diarrhea and/or constipation, and abdominal pain. The last decade saw an increase in the number of diagnosed SIBO cases, which is primarily due to the availability of noninvasive breath tests that facilitate the diagnostic process but are not considered as a gold diagnostic standard. The mainstay of SIBO management involves modulation of the small intestine microbiota in a manner that leads to improvement of symptoms. For this purpose, antibiotics are recommended, and recently, the eubiotic rifaximin has been shown to be effective against SIBO. Other biotic agents include selected probiotic strains exhibiting beneficial effects, as confirmed via breath tests. However, further randomized, placebo-controlled studies are needed to verify the efficacy of biotics.

## Figures and Tables

**Table 1:** Recommended preparation for breath testing.

Period before the Breath Test	Drugs/Activities to Be Avoided
4 weeks	Oral or intravenous antibiotics Prokinetic agents
2 weeks	Probiotics
1 week	Proton pump inhibitors
48 h	Motility regulators: loperamide, metoclopramide, trimebutine
24 h	Alcohol Fiber (particularly non-soluble fiber)
12 h	Oral food intake (only water is allowed)
The morning on the day of the test	Smoking Physical exertion Food Regularly used medications are allowed

## Data Availability

Not applicable.
